# Report of the Committee Appointed to Arrange for the Meeting of the International Medical Congress in America in 1887

**Published:** 1885-10

**Authors:** John V. Shoemaker

**Affiliations:** Secretary of the Committee of Arrangements


### REPORT OF THE COMMITTEE APPOINTED TO ARRANGE FOR THE MEETING OF THE INTERNATIONAL MEDICAL CONGRESS IN AMERICA, IN 1887.

At the regular annual meeting of the American Medical Association, held in New Orleans, in April, 1885, the following resolutions were adopted:

1. "*Resolved*, That the Committee appointed by this Association, to arrange for the meeting of the International Medical Congress in America, in 1887, be enlarged by the addition of thirty-eight members, one from each State and Territory, the District of Columbia, the Army, Navy, and Marine Hospital Service, to be appointed by the Chairman at this meeting, and that the Committee, thus enlarged, shall proceed at once to review, alter, and amend the motions of the present Committee as it may deem best."

(This resolution was amended by the provision, "that the members of the Committee should be selected by the respective State delegations.")

2. "*Resolved*, That the Committe appointed in pursuance of a resolution adopted by this Association, April 30, 1885, to constitute an addition to the original Committee of eight previously appointed to invite and make arrangements for the meeting of the International Medical Congress, to be held in Washington, D. C., in 1887, be, and the said Committee is, hereby authorized and empowered to select a Chairman and a Secretary, and to fill all vacancies that may occur by death or inability to attend in the Committee, and to appoint the officers of the Congress."

The following is a list of the Committee enlarged in accordance with the first resolution as amended:

W. E. Anthony, M. D., Providence, R. I.; G. Baird, M. D., Wheeling, W. Va.; Robert Battey, M. D., Rome, Ga.; F. W. Beard, M.D., Vincennes, Ind.; *J. S. Billings, M. D., U. S.

*Resigned.

Army, Washington, D.C.; *J. M. Browne, M. D., U. S. Navy, Washington, D. C.; L. P. Bush, M. D., Wilmington, Del.; H. F. Campbell, M. D., Augusta, Ga.; R. Beverly Cole, M. D., San Francisco, Cal.; E. P. Cook, M. D., Mendota, Ill.; W. C. Dabney, M. D., Charlottesville, Va.; Charles Denison, M. D., Denver, Col.; W. E. Duncan, M. D., Ellendale, Dakota Ter.; J. W. Dupree, M. D., Baton Rouge, La.; Ellsworth Eliot, M.D, New York City; *G. J. Englemann, M. D., St. Louis, Mo.; N. F. Essig, M. D., Plattsburg, Mo.; *Austin Flint, M. D., LL. D., New York City; E. P. Frazer, M. D., Portland, Oregon; George F. French, M. D., Minneapolis, Minn.; A. Y. P. Garnett, M. D., Washington, D. C.; S. C. Gordon, M. D., Portland, Me.; J. W. S. Gouley, M. D., New York City; F. M. Gunnell, M. D., U. S. Navy, Washington, D. C.; John B. Hamilton, M. D., U. S. Marine Hospital Service, Washington, D. C.; *I. M. Hays, M. D., Philadelphia, Pa.; *C. Johnston, M. D., Baltimore, Md.; George A. Ketchum, M. D., Mobile, Ala.; R. A. Kinloch, M. D., Charleston, S. Ca.; D. A. Linthicum, M. D., Helena, Ark.; John S. Lynch, M. D., Baltimore, Md.; J. J. McAchran, M. D., Laramie City, Wyom. Ter.; J. W. McLaughlin, M. D. Austin, Texas; R. C. Moore, M. D., Omaha, Neb.; Robert Murray, M. D., U. S. Army, Washington, D. C.; R. Murray, M. D., Moultrie, Fla.; J. W. Parsons, M. D., Portsmouth, N. H.; William Pierson, M. D., Orange, N. J.; N. J. Pitman, M. D., Tarboro, N. Ca.; *L. A. Sayre, M. D., New York City; X. C. Scott, M. D., Cleveland, O.; *Nicholas Senn, M. D., Milwaukee, Wis.; John V. Shoemaker, M. D., Philadelphia, Pa.; F. L. Sim, M. D., Memphis, Tenn.; A. R. Smart, M. D., Hudson, Mich.; D. W. Stormont, M. D., Topeka, Kan.; J. M. Taylor, M. D., Corinth,

*Resigned.

Miss.; E. F. Upham, M. D., West Randolph, Vt.; W. H. Wathen, M. D., Louisville, Ky.; W. Watson, M. D., Dubuque, Iowa; W. C. Wile, M. D., Sandy Hook, Conn.; A. H. Wilson, M. D., Boston, Mass.

At an informal meeting of the Committee, held at New Orleans during the session of the American Medical Association, in April, 1885, Dr. R. Beverly Cole, of San Francisco, Cal., was elected temporary Chairman, and Dr. John V. Shoemaker, of Philadelphia, Pa., was elected temporary Secretary.

The Committee held its first regular meeting at Chicago, Ill., on June 24th and June 25th, 1885, for the purposes of organization and the transaction of the business committed to it by the American Medical Association.

In order to facilitate the holding of meetings in different sections of the country, the Committee deemed it advisable to select a Vice-Chairman, in addition to a Chairman and a Secretary.

The following named members were present at the meeting held in Chicago:

G. Baird, M. D., Wheeling, W. Va.; Robert Battey, M. D., Rome, Ga.; F. W. Beard, M. D., Vincennes, Ind.; *J. S. Billings, M. D., Washington, D. C.; R. Beverly Cole, M. D., San Francisco, Cal.; E. P. Cook, M. D., Mendota, Ill.; W. E. Duncan, M. D., Ellendale, Dakota Ter.; Ellsworth Eliot, M. D., New York City; N. F. Essig, M. D., Plattsburg, Mo.; G. F. French, M. D., Minneapolis, Minn.; A. Y. P. Garnett, M. D., Washington, D. C.; John B. Hamilton, M. D., Washington, D. C.; *I M. Hays, M. D., Philadelphia, Pa.; George A. Ketchum, M. D., Mobile, Ala.; D. A. Linthicum, M. D., Helena, Ark.; John S. Lynch, M. D., Baltimore, Md.; J. W. McLaughlin, M. D., Austin, Texas; X. C. Scott, M. D., Cleveland, O.; * Nicholas Senn,

*Resigned.

M. D., Milwaukee, Wis.; John V. Shoemaker, M. D., Philadelphia, Pa.; F. L. Sim, M. D., Memphis, Tenn.; A. R. Smart, M. D., Hudson, Mich.; D. W. Stormont, M. D., Topeka, Kan.; E. F. Upham, M. D., West Randolph, N. Y.; W. Wathen, M. D., Louisville, Ky.; W. Watson, M. D., Dubuque, Iowa; A. H. Wilson, M. D., Boston, Mass.

The resignation of Dr. Austin Flint, of New York, as a member of the Committee, was presented and accepted. Dr. J. W. S. Gouley, of New York, was elected to fill the vacancy, and took his seat with the Committee. The Committee then organized, a majority of its members being present, by the election of the following officers:

*Chairman*, Dr. R. Beverly Cole, San Francisco, Cal.

*Vice-Chairman*, Dr. John S. Lynch, Baltimore, Md.

*Secretary*, Dr. John V. Shoemaker, Philadelphia, Pa.

After the organization of the Committee, the number of members necessary for a quorum for future meetings was fixed at fifteen.

The following preamble and resolution were adopted, to apply to future meetings of the Committee.

"WHEREAS, It is expedient that the meetings of this Committee shall represent, as far as practicable, the profession of all portions of our country,

"*Resolved*, That any member of this Committee who may be unable to attend a meeting, shall be empowered to send as his proxy for the meeting any member of the American Medical Association, in good professional standing and a resident of his State or a member of his Government Department."

In the course of the meeting in Chicago, on June 24th and 25th, 1885, a plan of organization of the Congress was adopted, certain officers of the Congress were appointed, in accordance with the instructions received from the American Medical Assoation, and the Committee adjourned.

The revised rules for the organization of the Congress and a revised list of officers will be presented with this report.

In August, 1885, the Chairman, the Vice-Chairman and the Secretary, after consultation and communication with members, called a meeting of the committee, to be held in New York City, September 3d, 1885, for the purposes of completing the revision of the rules and the filling of certain vacancies in the list of officers of the Congress.

The committee met in New York City, September 3d, 1885, the following named members being present:

Drs. G. Baird, Robert Battey, L. P. Bush, R. Beverly Cole, W. C. Dabney, Ellsworth Eliot, A. Y. P. Garnett, S. C. Gordon, J. W. S. Gouley, J. B. Hamilton, George A. Ketchum, R. A. Kinloch, D. A. Linthicum, John S. Lynch, R. C. Moore, William Pierson, N. J. Pitman, L. A. Sayre, X. C. Scott, John V. Shoemaker, F. L. Sim, E. F. Upham, W. H. Wathen, W. C. Wile, A. H. Wilson.

The following named members were represented by proxies.

Dr. E. P. Cook by Dr. N. S. Davis, proxy.

Dr. A. R. Smart, by Dr. William Brodie, proxy.

Dr. J. M. Taylor, by Dr. E. P. Sale, proxy.

The committee was called to order at 12 M., September 3d, 1885, by the Chairman, Dr. R. Beverly Cole.

The resignation of Dr. L. A. Sayre, of New York, as member of the committee, was received and accpted, and Dr. A. Flint, Jr., of New York, was elected to fill the vacancy, and took his seat with the committee. The resignation of Dr. Sayre was caused solely by ill health.

The following "Rules" were unimously adopted:

RULES.

1. The Congress shall consist of members of the regular profession of medicine, who shall have inscribed their names on the register, and shall have taken out their tickets of admission; and of such other scientific men as the Executive committee of the Congress may see fit to admit.

2. The dues for members of the Congress shall be ten dollars each for members residing in the United States.

There shall be no dues for members residing in foreign countries.

Each member of the Congress shall be entitled to receive a copy of the "Transactions" for 1887.

3d. The Congress shall be divided as follows, into seventeen sections:

I. General Medicine.

II. General Surgery.

III. Military and Naval Surgery.

IV. Obstetrics.

V. Gynæcology.

VI. Therapeutics and Materia Medica.

VII. Anatomy.

VIII. Physiology.

IX. Pathology.

X. Diseases of Children.

XI. Ophthalmology.

XII. Otology and Laryngology.

XIII. Dermatology and Syphilis.

XIV. Public and International Hygiene.

XV. Collective Investigation, Nomenclature, Vital Statistics, and Climatology.

XVI. Psochological Medicine and Diseases of the Nervous System.

XVII. Dental and Oral Surgery.

4. The general meetings of the Congress shall be for the transaction of business and for addresses and communications of general scientific interest.

5. Questions and topics that have been agreed upon for discussion in the sections shall be introduced by members previously designated by the titular officers of each section. Members who shall have been appointed to open discussions, shall present, in advance, statements of the conclusions which they have formed as a basis for debate.

6. Brief abstracts of papers to be read in the sections shall be sent to the secretaries of the proper sections on or before April 30, 1887. These abstracts shall be treated as confidential communications, and shall not be published before the meeting of the Congress.

Papers relating to topics not included in the list of subjects proposed by the officers of the sections may be accepted after April 30, 1887; and any member wishing to introduce a topic not on the regular lists of subjects for discussion, shall give notice of the same to the Secretary-General, at least twenty-one days before the opening of the Congress, and such notices shall be promptly transmitted by the Secretary-General to the Presidents of the proper sections. The titular officers of each section shall decide as to the acceptance of such proposed communications and the time for their presentation.

7. All formal addresses, scientific communications and papers presented, and scientific discussions held at the general meetings of the Congress, shall be promptly given in writing to the Secretary-General; and all papers presented and discussions held at the meetings of the sections shall be promptly given in writing to the Secretaries of the proper sections,

No communication shall be received which has already been published, or read before a society.

The Executive Committe, after the final adjournment of the Congress, shall direct the editing and the publication of its "Transactions," and shall have full power to publish the papers presented and the discussions held thereon, either in full, in part, or in abstract, as, in the judgment of the committee, may be deemed best.

8. The official languages of the Congress shall be English, French and German.

In the meetings of the sections no member shall be allowed to speak for more than ten minutes, with the exceptions of the readers of papers and those who introduce subjects for discussion, who may each occupy twenty minutes.

9. The rules and programmes shall be published in English, French and German.

Each paper and address shall be printed in the "Transactions" in the language in which it was presented, and preliminary abstracts of papers and addresses also shall be printed, each in the language in which it is to be delivered.

All discussions shall be printed in English,

10. The President of the Congress, the Secretary-General, the Treasurer, the Chairman of the Finance Committee and the Presidents of the sections, shall together constitute an Executive Committee of the Congress, which committee shall direct the business of the Congress, shall authorize all expenditures for the immediate purposes of the Congress, shall supervise and audit the accounts of the Treasurer, and shall fill all vacancies in theoffices of the Congress and of the sections. This Committee shall have power to add to its membership, but the total number of members shall not exceed thirty. A number equal to one-third of the members of the Committee shall constitute a quorum for the transaction of business.

11. The officers of the Congress shall be a President, Vice-Presidents, a Secretary-General, four Associate Secretaries, one of whom shall be the French Secretary, and one of whom shall be the German Secretary, a Treasurer, and the Chairman of the Finance Committee.

12. The officers of each section shall be a President, Vice-Presidents, Secretaries, and a Council.

13. The officers of the Congress and the officers of the sections shall be nominated to the Congress at the opening of its first session.

14. The Executive Committee shall, at some convenient time before the meeting of the Congress, prepare a list of foreign Vice-Presidents of the Congress and foreign Vice-Presidents of the Sections, to be nominated to the Congress at the opening of its first session.

15. There shall be a standing Committee on Finance, composed of one representative from each State and Territory, the District of Columbia, the Medical Department of the Army, the Medical Department of the Navy, and the Marine Hospital Service.

The Chairman of the Finance Committee shall report to the Executive Committee of the Congress.

Each member of the Finance Committee shall appoint a local Finance Committee for his State, Territory, District, or Government Department, consisting of one or more members from each Government Department or Congressional District.

Each local Finance Commmitte shall report through its Chairman to the Chairman of the Finance Committee of the Congress.

Dr. S. C. Gordon, of Maine, recalled his withdrawal from the Congress, which action was accepted by the Committee.

The following named gentlemen were elected to fill vacancies in the Committee of Arrangements: Dr. J. K. Bartlett, Wisconsin; Dr. J. H. Baxter, U. S. Army; Dr. George Goodfellow, Arizona; Dr. Henry Leffman, Pennsylvania; Dr. John Morris, Maryland; Dr. J. R. Tipton, New Mexico; Dr. Thomas J. Turner, U. S. Navy.

The following resolution was adopted:

*Resolved*, That the representative or representatives in this Committee from each State, Territory, or Government Department, shall organize the Financial Committees in their respective States, Territories, or Government Departments.

It was decided that no person should occupy more than one position in the organization of the Congress.

It was also decided that, in the published lists of the Officers of the Congress, the names of the Vice-Presidents and Secretaries of the Congress, and the Vice-Presidents, Secretaries, and members of Councils of the Sections, should be arranged alphabetically.

### OFFICERS OF THE CONGRESS.

#### PRESIDENT.

Austin Flint, M. D., LL. D., New York.

#### VICE-PRESIDENTS.

W. O. Baldwin, M. D., Alabama; H. I. Bowditch, M. D., Massachusetts; William Brodie, M. D., Michigan; Henry F. Campbell, M. D., Georgia; W. W. Dawson, M D., Ohio; R. Palmer Howard, M. D., Canada; E. M. Moore, M. D., New York; Tobias G. Richardson, M.D., Louisiana; Lewis A. Sayre, M. D., New York; J. M. Toner, M. D., District of Columbia; The President of the American Medical Association; The Surgeon-General of the United States Army; The Surgeon-General of the United States Navy; The Supervising Surgeon-General of the United States Marine Hospital Service.

#### SECRETARY-GENERAL.

Nathan S. Davis, M. D., LL. D., Chicago, Illinois.

#### TREASURER.

E. S. F. Arnold, M. D., M. R. C. S., New York.

CHAIRMAN OF THE FINANCE COMMITTEE.

Frederick S. Dennis, M. D., M. R. C. S., New York.

EXECUTIVE COMMITTEE OF THE CONGRESS.

GENERAL OFFICERS.

Austin Flint, M. D., LL. D., President of the Congress.

Nathan S. Davis, M. D., LL. D., Secretary-General.

E. S. F. Arnold, M. D., M. R. C. S., Treasurer.

Frederick S. Dennis, M. D., M. R. C. S., Chairman of the Finance Committee.

PRESIDENTS OF THE SECTIONS.

*General Medicine.*—Abram B. Arnold, M. D., Professor of Clinical Medicine, Baltimore, Md.

*General Surgery.*—William T. Briggs, M. D., Professor of Surgery, Nashville, Tenn.

*Military and Naval Medicine and Surgery.*—Henry H. Smith, M. D., formerly Professor of Surgery and Surgeon-General of Pennsylvania, Philadelphia, Pa.

*Obstetrics.*—DeLaskie Miller, Ph. D., M. D., Professor of Obstetrics, Chicago, Ill.

*Gynæcology.*—Robert Battey, M. D., Gynæcologist, Rome, Ga.

*Therapeutics and Materia Medica.*—F. H. Terrill, M. D., Professor of Therapeutics, San Francisco, Cal.

*Anatomy.*—William H. Pancoast, M. D., Professor of General, Descriptive, and Surgical Anatomy, Philadelphia, Pa.

*Physiology.*—John C. Dalton, M. D., Professor of Physiology, New York, N. Y.

*Pathology.*—E. O. Shakespeare, M. D., General Pathologist, Philadelphia, Pa.

*Diseases of Children.*—J. Lewis Smith, M. D., Professor of Diseases of Children, New York, N. Y.

*Ophthalmology.*—A. W. Calhoun, M. D., Professor of Ophthalmology and Otology, Atlanta, Ga,

*Otology and Laryngology,*—S. J. Jones, M. D., LL. D., Professor of Ophthalmology and Otology, Chicago, Ill.

*Dermatology and Syphilis.*—A. R. Robinson, M. D., Vice-President American Dermatological Society, New York, N. Y.

*Public and International Hygiene.*—Joseph Jones, M. D., Professor of Chemistry and Clinical Medicine, Ex-President Board of Health, New Orleans, La.

*Co lective Investigation, Vital Statistics and Climatology.*—Henry O. Marcy, A.. M., M. D., Boston, Mass.

*Psychological Medicine and Nervous Diseases.*—John P. Gray, M. D., LL. D., Professor of Psychological Medicine and Medical Jurisprudence, Utica, N. Y.

*Dental and Oral Surgery.*—Jonathan Tafft, M. D., Professor of Dental and Oral Surgery, Cincinnati, O.

LOCAL COMMITTEE OF ARRANGEMENTS.

(With power to increase their number.)

A. Y. P. Garnett, M. D., Chairman, Dist. of Columbia.

The Surgeon-General U. S. Army.

The Surgeon-General U. S. Navy.

The Supervising Surgeon-General U. S. Marine Hospital Service.

J. H. Baxter, M. D., District of Columbia.

C. H. A. Kleinschmidt, M. D., District of Columbia.

N. S. Lincoln, M. D., District of Columbia.

J. M. Toner, M. D., District of Columbia.

Lists of Vice-Presidents, Secretaries and Councilmen for each Section were named by the Committee of Arrangements, but as it was not practicable to ascertain at once who would accept the places assigned them, or who of those who had been announced in the medical press as declining to accept positions before the present rules and organization had been adopted as given above, might wish to withdraw such declination, the final adjustment of these offices was referred to the Executive Committee of the Congress, and all correspondence in relation thereto was transferred to the Secretary-General of the Congress.

On motion, the Committee of Arrangements adjourned, subject to the call of the Chairman of the Committee.

JOHN V. SHOEMAKER,
Secretary of the Committee of Arrangements

